# Eradication of *Candida albicans* persister cell biofilm by the membranotropic peptide gH625

**DOI:** 10.1038/s41598-020-62746-w

**Published:** 2020-04-01

**Authors:** Emilia Galdiero, Elisabetta de Alteriis, Antonino De Natale, Angela D’Alterio, Antonietta Siciliano, Marco Guida, Lucia Lombardi, Annarita Falanga, Stefania Galdiero

**Affiliations:** 1 Department of Biology, University of Naples “Federico II”, via Cinthia, 80100 Naples, Italy; 2 Department of Pharmacy, University of Naples “Federico II”, Via Mezzocannone 16, 80134 Naples, Italy; 3 Department of Agricultural Science, University of Naples Federico II, Via Università 100, 80055 Portici, Naples, Italy

**Keywords:** Infection, Medicinal chemistry

## Abstract

Biofilm formation poses an important clinical trouble due to resistance to antimicrobial agents; therefore, there is an urgent demand for new antibiofilm strategies that focus on the use of alternative compounds also in combination with conventional drugs. Drug-tolerant persisters are present in *Candida albicans* biofilms and are detected following treatment with high doses of amphotericin B. In this study, persisters were found in biofilms treated with amphotericin B of two clinical isolate strains, and were capable to form a new biofilm *in situ*. We investigated the possibility of eradicating persister-derived biofilms from these two *Candida albicans* strains, using the peptide gH625 analogue (gH625-M). Confocal microscopy studies allowed us to characterize the persister-derived biofilm and understand the mechanism of interaction of gH625-M with the biofilm. These findings confirm that persisters may be responsible for *Candida* biofilm survival, and prove that gH625-M was very effective in eradicating persister-derived biofilms both alone and in combination with conventional antifungals, mainly strengthening the antibiofilm activity of fluconazole and 5-flucytosine. Our strategy advances our insights into the development of effective antibiofilm therapeutic approaches.

## Introduction

Biofilms are established by microbial cells that attach to inert or living surfaces and gather in a polymeric matrix of their own synthesis forming sessile communities resistant to antimicrobial agents and showing a lifestyle distinct from that of planktonic cells^1,2^. Biofilms provide a protected growing, allowing cell survival in a hostile environment and show emergent properties that are not foreseeable from the investigations on planktonic cells^3^. Moreover, biofilms often are not caused by a single-species population of microbes but by mixtures of species of bacteria or fungi and develop on inert surfaces such as medical devices, on living tissues, and also on fragments of dead tissues^4^. Conventional antifungal therapies typically contrast the symptoms of planktonic cells released from the biofilm, but fail to kill the biofilm itself; thus, after cycles of antifungal therapy, biofilm infections are typically recurring until surgically removed^5^.

In this work, we focused on biofilms formed by *Candida* spp, which is regarded as an important source of nosocomial systemic infections related to mortality rates up to 40%, with *Candida albicans* being the most widespread species^6^. *Candida* spp form biofilms on various surfaces, thus being responsible for medical device-associated infections. The high resistance of *Candida* biofilms to conventional treatments is associated to several issues: (i) drug sequestration by the matrix; (ii) up-regulation of drug efflux pumps; (iii) presence of multidrug-tolerant persister cells^7^.

Persistence is the ability of a small part of the clonal population to survive exposure to high doses of antimicrobial treatment^8^. Persister cells present a particular case of tolerance (able to survive a transient exposure to antibiotics) and constitute a subpopulation (on average less than 1%) that is killed at a slower rate than susceptible cells^8^. Persisters have been initially detected in mature biofilms of *C. albicans*^9^ and then in several other *Candida* spp^10^.

The slower rate of killing of the persistent subpopulation is non-heritable; as a matter of fact, when the original population is treated with an increasing amount of drug, a biphasic killing pattern is observed, by which the majority of the population is killed, while a small portion is able to survive; in addition, if persistent cells are isolated, regrown and repetitively treated with high drug concentrations, the same response to the drug will be observed as in the original population. Persisters are apt to enter into a metabolically inactive state as a response to antifungal stress; as soon as the antimicrobial treatment ends, persisters are able to revive and repopulate the biofilm. Therefore, the occurrence of recalcitrant infections has been correlated to persistence^10^.

Currently, compounds on the market are able to fully eradicate fungi *in vitro*; however, they are not always effective in a clinical setting, where immunomodulation and device implantation put patients at higher threat of fungal infections^11^. Treatments include the use of polyenes (such as amphothericin B, AmphB), azoles (such as fluconazole, FLC), echinocandins, and 5-flucytosine (5-FC)^12^.

Persister cells of *C. albicans* are typically detected using AmphB as fungicidal drug; this is essentially because AmphB acts through sequestration of ergosterol, which renders development of resistance quite rare, which increases the chance that the surviving colonies are persister cells and not resistant isolates^13^.

A recent approach to deal with biofilms and multidrug-resistant infections has involved the exploitation of antimicrobial peptides (AMPs) as alternative compounds. Several AMPs were shown to have enhanced antibiofilm activities compared to conventional antibiotics^14–17^. AMPs are short cationic sequences containing both hydrophobic and hydrophilic amino acids, yielding amphipathic structures and widely diffused both in the animal and plant world, with activities against a broad spectrum of microorganisms^18–21^. Their mechanism of action mainly involves the pore-forming activity, which also reduces the possibility to develop bacterial resistance; although there are also non-lytic AMPs with intracellular targets^20^. As a matter of fact, among AMPs, there are membranotropic peptides, which are able to interact, transiently and locally disrupt membrane bilayers and often behave as cell penetrating peptides (CPPs)^21^. It is extremely interesting to probe the eventual enhancement of activity of drugs that act on the membrane or inside the cell when used in combination with CPPs. gH625 is a member of this class of peptides marked by the presence of a high amount of alanine, glycine and leucine residues responsible of its innate conformational flexibility and ability to adopt diverse secondary structures in different environments, and aromatic residues involved in the preferential localization of the peptide at the membrane interface^22–28^. gH625 is also an effective drug delivery carrier thanks to its ability to interact with membrane bilayers^22,25,26,29,30^ without displaying toxic effect on mammalian cells^31,32^.

Recently, the antimicrobial and antibiofilm activities of a gH625 analogue achieved adding a sequence of lysine residues at its C-terminus, responsible of specific interactions with the negative charges of bacterial membranes, has been examined^33,34^. The peptide gH625-M showed a low activity against planktonic cells, while it impaired formation of polymicrobial biofilms of clinical isolates of *Candida tropicalis/Serratia marcescens* and *Candida tropicalis/Staphylococcus aureus* and influenced the biofilm architecture, interfering with cell adhesion and polymeric matrix; moreover, it eradicated the long-term polymicrobial biofilms established on silicone surfaces^33^. The mechanism of action is not fully undisclosed; nonetheless, the experimental results point to the fact that gH625 does not form pores in the target membrane but it acts on the biofilm structure and thus this may justify its low activity against planktonic cells^33^. Molecules with low activity against planktonic cells may be used at lower concentrations compared to the MIC and likely they will interfere with the biofilm life-style rather than kill cells^35^.

In this work, we used two analogues of gH625, namely the gH625 monomer (gH625-M) and the gH625-dimer (gH625-D), to dissect the role played by the charges, the dimerization and the hydrophobic domain in the interaction with *C. albicans* biofilms. Once established the antibiofilm activity of the two analogues towards mature *Candida* biofilms, the most effective molecule (gH625-M) was used to combat the persister-derived biofilm from a reference strain and a clinical isolate. Indeed, the formation of the persister-derived biofilm was investigated to simulate the re-colonization of a surface, as occurring *in vivo* following the antifungal therapy.

Furthermore, since combination therapy may represent a promising strategy to extend the efficacy of current drugs against biofilms^36,37^, we investigated the possible synergistic activity of gH625-M in combination with common antifungal drugs on eradication of the persister-derived *C. albicans* biofilms.

## Materials and Methods

### Synthesis of gH625-M, gH625-D, and FITC-gH625-M

Peptides were synthetized using the Fmoc solid-phase method^38^, using a Rink Amide MBHA (0.54 mmol/g) resin and through consecutive deprotection (30% piperidine in dimethylformamide (DMF), 2 × 10 min) and coupling steps. As for the coupling steps, the first one was performed in presence of 4 equivalents of amino acid, 4 equivalents of dicyclohexylcarbodiimide (DIC) and 4 equivalents of oxymapure; while the following couplings were performed with 4 equivalents of amino acid, 4 equivalents of 1-[Bis(dimethylamino)methylene]-1H-1,2,3-triazolo[4,5-b]pyridinium3-oxidhexafluorophosphate (HATU) and 8 equivalents of N,N-Diisopropylethylamine (DIPEA). A solution of trifluoroacetic acid (TFA) (TFA: H_2_O: thioanisole: ethanedithiol: anisole 85:5:5:3:2 v/v) for 5 h at room temperature was used for side chain deprotection and cleavage from the resin. After deprotection, peptides were precipitated in cold ethylic ether and analysis of the crudes was performed by liquid chromatography-mass spectrometry (LC–MS) using a gradient of acetonitrile (0.1% TFA) in water (0.1% TFA) from 20 to 80% in 15 min. Purifications were accomplished by preparative reverse phase high performance liquid chromatography (RP-HPLC). All purified peptides were attained with good yields (50–60%). Table 1 shows the sequences of the synthesized peptides. The dimer gH625-D was obtained by oxidation. Peptide oxidation was performed in ammonium bicarbonate 0.1 M pH 8.2 overnight. After oxidation the obtained compound was further purified by RP-HPLC.

**Table 1:** Peptides sequences.

Peptide	Sequence
gH625-M	NH_2_-HGLASTLTRWAHYNALIRAFGGGKKKK-CONH_2_
FITC-gH625-M	FITC-HGLASTLTRWAHYNALIRAFGGGKKKK-CONH_2_
gH625-D	NH_2_-HGLASTLTRWAHYNALIRAFGCGKKKK-CONH_2_ NH_2_-HGLASTLTRWAHYNALIRAFGCGKKKK-CONH_2_

For confocal laser scanner microscope (CLSM) analysis, the peptide gH625-M was conjugated with fluorescein isothiocyanate (FITC). FITC was introduced at the N-terminal side, the reaction was performed on the resin bound peptides in presence of 5 equivalents of FITC and 5 equivalents of triethylamine (TEA) in DMF overnight. The crude labelled peptide was cleaved from the resin, purified by HPLC and characterized as reported above.

All peptide identities were confirmed by mass spectroscopy using LTQ-XL Thermo Scientific instrument before use.

### *Candida albicans* strains and culture conditions

*C. albicans* strain ATCC 90028 and an isolate (C1) obtained from samples collected during a previous surveillance study performed by our group^39^, were routinely maintained in Sabouraud dextrose agar (1% yeast extract, 1% peptone, 4% glucose, 1% agar) at 4 °C. To prepare a standard cell suspension, a single colony was inoculated into Tryptone Soya Broth (TSB) (Oxoid) medium with 1% of glucose and incubated overnight at 37 °C at 200 rpm. For antifungal activity *in vitro*, fungal cells were harvested by centrifugation, washed twice in PBS and resuspended in TSB medium with 1% glucose to 1 × 10^6^ colony forming units (CFU) mL^−1^. For biofilm growth, fungal cells were harvested by centrifugation, washed twice in PBS (pH 7.2) and resuspended with RPMI 1640 (w/o glutamine) (Lonza, Switzerland) to 1 × 10^6^ CFUmL^−1^. For CFU assay, serial dilutions were performed by the standard procedure, and spread onto plates containing yeast extract peptone dextrose agar medium (YPDA) (1% yeast extract, 2% bactopeptone, 2% glucose, 2% agar). Plates were incubated at 37 °C for 48 h before counting the colonies.

### Susceptibility of planktonic cells to conventional antifungal drugs and gH625 analogues

Determination of the minimum inhibition concentration (MIC) was performed according to the Clinical and Laboratory Standards Institute (CLSI) guideline 2008 M27-A3^40^. Briefly, the MIC was performed in 96-well plate and the suspension of 100 μL of TBS 1% glucose containing 1 × 10^6^ cells/mL was inoculated in each well and incubated for 24 h at 37 °C. Gradients of concentrations used were from 0.01 to 10 µg mL^−1^ for AmphB (European Pharmacopea Reference Standard), fluconazole (FLC) and 5-flucytosine (5-FC) (European Pharmacopea Reference Standard). gH625-M and gH625-D were used at concentrations ranging from 2.5 to 50 μM. The growth of each strain was measured by the turbidity method using an ELISA plate reader at 590 nm wavelength (SINERGY Ha BioTek). MIC_80_ was estimated as the concentration capable of inhibiting 80% of growth of each microbial pathogen.

### Biofilm formation, Minimal Biofilm Eradication Concentration (MBEC) determination of conventional fungicides and gH625 analogues

To allow biofilm formation, 100 μL of the suspensions of the two strains (1 × 10^6^ CFU mL^−1^) in RPMI medium were loaded into polystyrene 96-well microplate and incubated at 37 °C for 48 h. Wells were then washed three times with 200 μL PBS to remove non-adherent cells.

The Minimal Biofilm Eradication Concentration (MBEC) was determined by adding to the 48 h biofilm, scalar concentration of the conventional drugs, namely Amph B from 0.05 to 10 µg mL^−1^, FLC and 5-FC from 1 to 2500 µg mL^−1^. Stock solutions of drugs at a concentration of 2.5 mg mL^−1^ in DMSO 1.3% v/v were prepared; preliminary studies showed that DMSO is not toxic against *C. albicans* up to concentrations of 5% v/v (data not shown).

In the case of gH625-M and gH625-D concentrations ranging from 2.5 to 50 μM (7.5 to 150 µg mL^−1^) in the presence of RPMI medium were used. The microplate was incubated at 37 °C for 24 h and residual biofilm quantified. MBEC_80_ was estimated as the concentration capable of eradicating 80% of the fungal biofilm.

Biofilm quantification was performed determining cell metabolic activity using a 2, 3-bis (2-methoxy-4-nitro-5-sulfo-phenyl)-2H-tetrazolium-5 carboxanilide (XTT) reagent (Kit cell Counting Kit-8 Enzolife Science, Switzerland)^33^.

### Detection of persister cells in *Candida* biofilms

The quantification of persisters was performed as previously described by LaFleur *et al*.^9^. Briefly, the biofilms, grown as previously described, were challenged with AmphB buffered to pH 7 with 0.165 M morpholinopropanesulfonic acid (MOPS, Sigma) at concentrations ranging from 5 to 100 μg mL^−1^ for 24 h at 37 °C in RPMI medium. Biofilms were abundantly washed with PBS, then disrupted by scraping and vortexed vigorously for 30 s before serial dilution and plating. The number of surviving cells (persisters) was determined by CFU assay.

### Development of persister-derived biofilm

The small proportion of cells not susceptible to AmphB treatment (persisters) of both strains was used as inoculum to obtain a new biofilm in the 96-well microplate. Subsequently, after AmphB treatment, the wells were abundantly washed with PBS, and incubation was continued for 8 days during which 100 μL TSB-glucose medium was added to each well every 24 h. Biofilm mass was estimated every 48 h by CFU assay, as described above.

### Activity of gH625-M and synergistic effect with antifungal drugs on eradication of persister-derived biofilm

Eradication activity, expressed as MBEC_50_, of the three fungicides, AmphB, FLC and 5-FC, as well as of gH625-M on the persister-derived biofilms from the two strains was determined as described for the 48 h biofilms. The potential synergistic/antagonistic effects of the gH625-M/AmphB, gH625-M/FLC and gH625-M/5-FC combinations on the eradication of persister-derived biofilms were determined by checkerboard microdilution assays as described by Wei *et al*.^41^. Each well of the 96-well microtiter plate containing the biofilm was treated with serially diluted test agents in combination. The initial concentrations used to calculate MBEC_50_ ranged from 2.5 to 50 µM (7.5 to 150 µg mL^−1^) for gH625-M, from 1 to 2500 µg mL^−1^. FLC and 5-FC, and from 0.5 to 10 µg mL^−1^ for AmphB. For the combination experiments, the concentration used ranged from 2.5 to 20 µM (7.5 to 60 µg mL^−1^) for gH625-M, from 0.05 to 5 µg mL^−1^ for FLC, 5-FC and AmphB. The eradicating activity was evaluated by calculation of the fractional inhibitory concentration index (FICI); FICI for all the combinations was determined as [(minimum concentration of drug A in combination)/(minimum concentration of drug A alone)] + [(minimum concentration of drug B in combination)/(minimum concentration of drug B alone)]. FICI ≤ 0.5, 0.5 < FICI < 4, or FICI > 4, were indicative of synergistic, indifferent or antagonistic effect respectively^42^. All the experiments were repeated twice, and the data were expressed as arithmetic average.

### Confocal laser scanner microscope observations of persister-derived biofilm

Confocal laser scanning microscopy (CLSM) studies were carried out in order to examine the architecture of the biofilm and the viability of the cells before and after exposure to antifungal agents with inverted confocal laser-scanning microscope (Zeiss LSM700, with a Zen 2011 software) by capturing images at 40X and 63X. The biofilms were detected in three replicates, for each replicate 3 z-stacks were identified and randomly recorded. Biofilms formed on the bottom of a multiwell plate for high resolution microscopy (microplate 96 well uncoated, Ibidi) were washed with PBS and stained with 5 µM Syto 9 (Invitrogen) that penetrates both viable and nonviable cells, and 20 µM propidium iodide, a non-vital nuclear stain commonly used for identifying dead cells, for 15 min at room temperature, in the dark.

The images from stacks were captured at 0.47 µm intervals using a magnification 40X. The substratum area of the image stack was 1024 × 1024 pixel (640.174 × 640.174 μm), with the number of images in each stack varying according to the thickness of the biofilm. The open source image processing package Fiji^43^ (see also http://www.fiji.sc) was used to evaluate the area of all stacked CLSM images and to obtain 2D maximum intensity projections (MIPs) and for the creation of 3D images. The images have been previously converted to 8-bit and then resampled by using the tool Threshold^44–46^. *Comstat2*^47^, *3D Manager*^48^ tools were used to determine features (substrate coverage, volume, bio-volume, thickness, roughness,) of the biofilm in each Z-stacks, to reconstruct the three dimensional architecture of the mats.

To observe localization of gH625-M, the biofilm in the well was stained with 50 μL of 0, 25 mg mL^−1^ Calcofluor, and 2 µL of Concanavalin-A with Alexa 488 for *Candida* cells and extracellular polymeric substance (EPS), respectively. Then, the peptide labeled with FITC at a concentration of 25 μM was added to the biofilm and observation performed after 30 and 180 min.

The images from stacks were captured as previously described. The quantification of the peptide penetrated into the walls and inside the cells was measured through the realization of 5 subz-stacks, including the single *Candida* cells, at 0.32 µm intervals with 63X magnification Appropriate Region Of Interest (ROI) was created and applied to the individual images of the new z-stacks, isolating the regions of the wall from that of the inner part^49^. The regions of the walls and central parts of the cells were analyzed as described for total biofilm. The volume of each cell was then used to evaluate the presence of EPS in areas outside the cells. The calculations carried out on 20 *Candida* cells randomly identified, for each of the three replicates.

### Statistical analysis

Results are given as mean ± std. dev. Differences between samples and control group were determined by two-tailed Student’s t-test. One-way analysis of variance (ANOVA) and further statistical *post-hoc* comparisons with Tukey’s multiple comparison test were used to evaluate significance of differences among groups. Differences were considered significant when p < 0.05.

## Results

### Peptide design

The two analogues of gH625, namely gH625-M and gH625-D, were designed exploiting the host-guest technology^34,50^. The polar features of the host peptide enables good solubility in aqueous buffer of the guest peptide and allows the peptide to remain monomeric in low ionic strength solutions. Furthermore, the presence of four consecutive lysine residues assists the guest peptide in the binding to lipid bilayers. Briefly, we added a positively charged fragment of four lysine residues (KKKK) (Table 1) which should favour not only the interaction with the negatively charged cell membranes, but also enhance the solubility of the mainly hydrophobic peptide gH625; moreover, the presence of the GGG or the GCG sequence allowed us to evaluate the effect of multivalency on the interaction^34^. The cysteine was exploited to form the dimer, and guarantees that both the N-terminus of the monomeric units remain free for membrane interactions, which is key because gH625 was proved to enter the bilayer from its N-terminal side^51^. The GGG sequence was used in the monomeric peptide, the GCG for the dimeric form. Synthesis and structural characterization of gH625-M and gH625-D have been previously reported^34^.

### Susceptibility of *Candida albicans* planktonic cells and biofilms to conventional antifungal drugs and gH625 analogues

The antifungal activity of the three conventional drugs, namely AmphB, FLC and 5-FC, and of the two peptides (gH625-M and gH625-D) against planktonic cells of *C. albicans* strains ATCC 90028 and C1 was expressed as MIC_80_ values, and is reported in Table 2. Both strains resulted susceptible to the conventional drugs, especially to AmphB. Instead, the two peptides tested showed a quite low antifungal activity (>150 μg mL^−1^).

**Table 2:** MIC_80_ and MBEC_80_ values of AmphB, 5-FC, FLC, gH625-M, gH625-D against the two fungal strains.

*C. albicans* strain	Concentration (μg/mL)	AmphB	5-FC	FLC	gH625-M	gH625-D
ATCC 90028	MIC_80_	0.5	1.8	2.3	>150 (50 μM)	>150 (50 μM)
	MBEC_80_	3.5	>2000	>2000	75 (25 μM)	>150 (50 μM)
C1	MIC_80_	0.1	1.3	2.3	>150 (50 μM)	>150 (50 μM)
	MBEC_80_	4.5	>2000	>2000	120 (40 μM)	>150 (50 μM)

All data are reported as μg mL^−1^; for gH625-M and gH625-D also the molar concentration is reported in parenthesis.

The biofilm eradication ability of the three antifungals and of gH625 analogues against 48 h *Candida* biofilms was estimated by the XTT assay and results are shown in Table 2. Dose-dependent linear decrease of biofilm density was observed. Whereas AmphB was very effective in eradicating the biofilms of both strains, MBEC_80_ of FLC and 5-FC were higher than 2000 µg mL^−1^, showing that they are quite ineffective to combat already formed *Candida* biofilms. These findings are in agreement with what reported in literature^36^. Interestingly, the peptides were effective in eradication of biofilms of both strains, with the monomeric form showing a higher activity with respect to the dimeric one. In details, at doses of 75 and 120 μg mL^−1^ of gH625-M (corresponding to 25 and 40 μM, respectively) and higher than 150 μg mL^−1^ (corresponding to 50 μM) of gH625-D, 80% biofilm degradation was observed for both strains.

### Detection of persisters cells in *Candida albicans* biofilms

**Figure 1:**
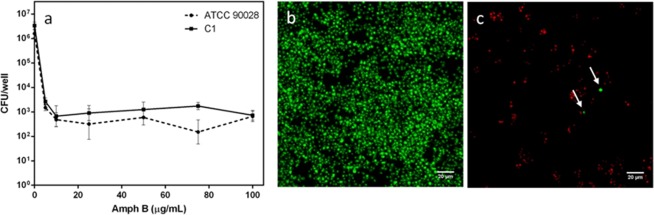
Detection of persisters in *Candida albicans* biofilms. (**a**) Survival of *Candida albicans* cells in biofilms of ATTC 90028 and C1 strain following treatment with AmphB at different concentrations. CLSM images of C1 biofilm before (**b**) and after (**c**) treatment with 100 µg mL^−1^ AmphB. In the untreated biofilm cells appear green (live), in the treated biofilm the majority of cells are red (dead). Arrows indicate the few persisters.

The presence of persister phenotypic variants of the wild type was indicated by a biphasic killing curve revealing a subpopulation of cells that survive high doses of antimicrobial. Both the strains of *C. albicans*, ATTC 90028 and C1, were able to form stable biofilms and in both biofilms, persisters were detected by a dose-dependent killing treatment with AmphB, as reported in Fig. 1. The majority of the biofilm population was killed at low concentrations with no statistical differences for both strains (p > 0.05), while the remaining cells were unaffected even by higher concentrations of the antifungal. Around 0.01–0.03% of the entire population appeared absolutely invulnerable to AmphB, indicating the presence of persisters in both biofilms. Live persister cells in the C1 strain biofilm could be observed by CLSM, after staining with Syto 9 and PI (Fig. 1b,c).

### Persister cells are able to develop a new biofilm *in situ*

Persister cells of both *Candida* strains, formed a new biofilm *in situ* (i.e. on the surface of the multi-well plate), when fresh medium was dispensed into the wells every 24 h, and plate incubation prolonged for 8 days.

**Figure 2:**
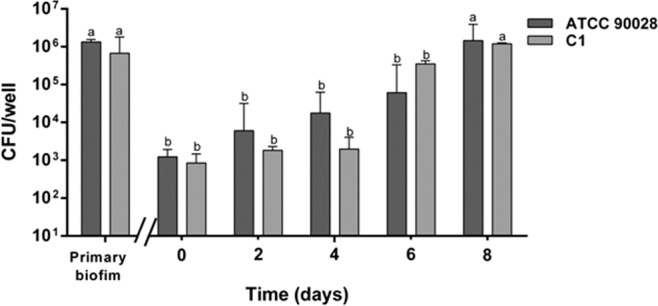
Development of a new biofilm from persisters. Viable cells increase during formation of the biofilm generating from cells of the primary biofilm survived at AmphB treatment at time = 0. Data with different letters (**a**,**b**) are significantly different (p < 0.05).

In Fig. 2, the progressive formation of the new biofilm is reported. The number of living cells estimated as CFU/well progressively increased, and reached the value of the primary biofilm after 8 days.

**Figure 3:**
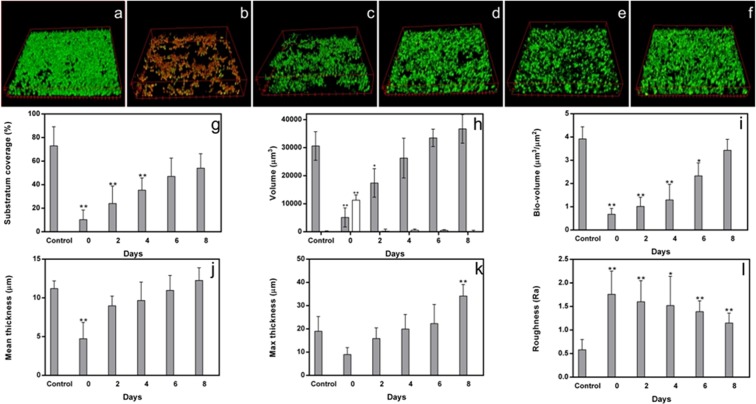
CLSM images (x400 magnification) of the primary biofilm of C1 strain before (**a**) and after AmphB treatment at time 0 (**b**) and the progressive formation of persister-derived biofilms after 2d (**c**), 4d (**d**), 6d (**e**), 8d (**f**) of incubation. In the graphs, substrate coverage (**g**), volume (**h**), bio-volume (**i**), mean thickness (panel j), maximal thickness (**k**), and roughness coefficient (**l**) of the C1 strain persister-derived biofilm are reported, as compared to the corresponding parameters of the primary biofilm (control). In panel h, white bar represents the volume of the dead cells. Significance noted as: *p < 0.05; **p < 0.01”.

The progressive formation of the persister-derived biofilm at the time intervals 0, 2, 4, 6, 8 days in the course of the experiment was also analyzed by CLSM (Fig. 3). In Fig. 3 it is possible to observe the change of the persister-derived biofilm features from 0 to 8 days, with a net increase in the viable cells (green) already after 2 days (Fig. 3, panel c) compared to the initial predominance of dead cells (red) (Fig. 3, panel b) following the AmphB treatment of the primary biofilm (Fig. 3, panel a).

Several structural parameters have been evaluated by CLSM analysis during persister-derived biofilm development, and compared to the primary biofilm: substrate coverage (panel g) indicating the percentage of surface colonization; volume (panel h); bio-volume (panel i), indicating the entire volume of the biofilm; biofilm mean (panel j) and maximal thickness (panel k); roughness coefficient (panel l), indicating the variation in biofilm mean thickness.

**Figure 4:**
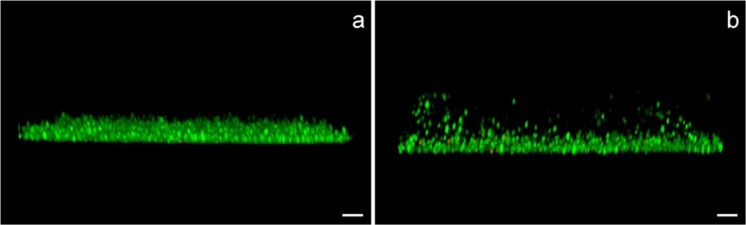
Biofilm profiles of the primary biofilm (**a**) and of the persister-derived biofilm at 8 d (**b**). In the latter, it is possible to visualize biofilm fragmentation. Bars correspond to 10 μm.

The first three parameters (Fig. 3, panels g,h,i), namely substrate coverage, volume and biovolume, are in agreement with the total viable count results (Fig. 2), and clearly show a progressive increase of the biofilm viable biomass during incubation. Following AmphB treatment, most of the biofilm was formed by dead cells (Fig. 3, panel b) and only persistent cells remained alive; after 2 days *Candida* cells had already reformed a biofilm and after 8 days the mass was even increased compared to the primary biofilm (control). The mean thickness shows the same trend. Indeed, the maximum thickness (Fig. 3, panel k) was considerably increased at 8 days due to an apparent diversification of the biofilm structure, presumably due to the presence of the aerial hyphae (Fig. 4).

Overall, the persister-derived biofilm appears less compact (higher thickness and roughness) compared to the primary one. In particular, the observed fragmentation could be attributed to the occurrence of aerial hyphae, possibly favouring the consequent dispersion of new cell units with a high potential of colonization.

### Persister-derived biofilms: eradication with gH625-M and combined effects of gH625-M and antifungals

The two persister-derived biofilms were treated with scalar concentration of gH625-M and the three antifungal drugs, AmphB, FLC and 5-FC. All tested compounds resulted efficient in the eradication of persister-derived biofilms, although MBEC_80_ was not achieved. Therefore, the MBEC_50_ values were calculated and reported in Table 3. The concentrations used to calculate MBEC_50_ ranged from 2.5 to 50 µM (7.5 to 150 µg mL^−1^) for gH625-M, from 1 to 2500 µg mL^−1^. FLC and 5-FC, and from 0.5 to 10 µg mL^−1^ for AmphB. Further, different combinations of gH625-M with AmphB, FLC and 5-FC were tested against *C. albicans* persister-derived biofilms, in order to prevent the development of drug resistance and to broaden their antimicrobial spectrum, reducing eventual toxic effects. For the combination experiments, the concentration used ranged from 2.5 to 20 µM (7.5 to 60 µg mL^−1^) for gH625-M, from 0.05 to 5 µg mL^−1^ for FLC, 5-FC and AmphB. C_AmphB_, C_5-FC_, C_FLC_ and C_gH625-M_ represent the minimum concentrations of each molecule to obtain 50% of eradication in combination experiments. The FICI values, were thus calculated considering the MBEC_50_ of the single drugs and gH625-M and are shown in Table 3.

**Table 3:** Effects of antifungals and gH625-M alone and in combination against the two *Candida albicans* persister-derived biofilms, evaluated by the fractional inhibitory concentration (FICI) model.

*C. albicans* strain	MBEC_50_ (μgmL^−1^)				FICI model	
	MBEC_AmphB_	C_AmphB_	MBEC_gH625-M_	C_gH625-M_	FICI	Interpretation
ATCC 90028	0.7	0.2	47.4 (15.9 μM)	11.3 (3.8 μM)	0.5	Synergy
C1	0.1	0.1	61.1(20.5 μM)	13.4 (4.5 μM)	0.8	No interaction
	**MBEC**_**5-FC**_	**C**_**5-FC**_	**MBEC**_**gH625-M**_	**C**_**gH625-M**_	**FICI**	**Interpretation**
ATCC 90028	>2000	0.2	47.4(15.9 μM)	11.3 (3.8 μM)	0.2	Synergy
C1	>2000	0.2	61.1(20.5 μM)	13.4 (4.5 μM)	0.2	Synergy
	**MBEC**_**FLC**_	**C**_**FLC**_	**MBEC**_**gH625-M**_	**C**_**gH625-M**_	**FICI**	**Interpretation**
ATCC 90028	>2000	0.1	47.4(15.9 μM)	14.9 (4.5 μM)	0.3	Synergy
C1	>2000	0.3	61.1(20.5 μM)	19.6 (6.6 μM)	0.3	Synergy

MBEC_50_ minimal biofilm‐eradication median concentration; MBEC_AmphB_, MBEC_5-FC_, MBEC_FLC_ and MBEC_gH625-M_ when used alone; C_AmphB_, C_5-FC_, C_FLC_ and C_gH625-M_ when used in combination. All data are reported as μg mL^−1^; for gH625-M also the molar concentration is reported in parenthesis.

gH625-M in combination with each antifungal showed a synergistic effect against the persister-derived biofilm at low doses of antifungal (FICI ≤ 0.5) (except in the case of C1 strain biofilm treated with gH625/AmphB), whereas the combinations gH625-M/antifungal did not show any synergistic action at higher doses (data not shown). Interestingly, the combination gH625-M/FC and gH625-M/5-FLC were very effective against biofilm, contrarily to the use of the same antifungals alone, also at high concentrations (Tables 2 and 3).

We hypothesized that the synergistic activity is likely due to the perturbation of the biofilm esopolymeric matrix and/or the walls of the *Candida* cells, by the peptide, which consequently favoured an enhanced penetration of FLC and 5-FC into the cells.

### Localization of FITC-gH625-M in *Candida* biofilm

**Figure 5:**
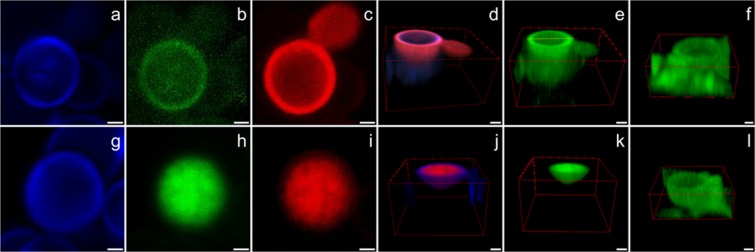
Cell details from C1 persister-derived biofilm after 30 min (**a**–**f**) and 180 min (**g**–**l**) from peptide treatment. Calcofluor-white dye (in blue: **a**,**g**), Concanavalin-A (in green: **b**,**h**), peptide (in red: **c**,**i**). 3D reconstruction of a *Candida* hemicell, to allow a better visualization of the peptide penetration inside the cells. After 30 minutes, the peptide penetrated mainly into the cell wall (**d**), after 180 minutes the peptide was found in a massive way inside the cells (**j**). Highlights of the EPS matrix in treated biofilms (**e**,**k**) and in the control (**f**,**l**). Bar scales correspond to 1 μm.

To determine whether the activity of gH625-M is correlated to an intracellular target once translocated into cytoplasm or to an effect on the EPS or the cell wall, the localization of gH625-M in the biofilm was analysed by CLSM, using the FITC-labeled peptide (Tables 4 and 5, Fig. 5).

**Table 4:** Structural parameters of *Candida* biofilm after treatment with gH625-M.

Minutes	Substratum coverage (%)	Bio-volume (μm^3^/μm^2^)	Mean thickness (μm)	Max thickness (μm)	Roughness (Ra)	Dye	Volume (%)
0	68.6 ± 8.6	3.3 ± 1.2	14.5 ± 2.1	25.3 ± 3.9	0.5 ± 0.1	Calcofluor	69.4 ± 8.6
						Concanavalin-A	30.6 ± 5.5
30	59.6 ± 10.3	1.3 ± 0.1	12.4 ± 2.0	20.6 ± 3.1	0.5 ± 0.1	Calcofluor	76.7 ± 8.6
						Concanavalin-A	14.4 ± 4.3
						FITC- gH625-M	8.9 ± 2.4
180	48.2 ± 9.6	1.3 ± 0.1	10.8 ± 1.5	19.2 ± 2.0	0.6 ± 0.1	Calcofluor	78.6 ± 5.9
						Concanavalin-A	9.2 ± 1.2
						FITC- gH625-M	12.2 ± 2.1

**Table 5:** Percentage of peptide presents in cell wall, cell interior and in the external EPS.

Peptide (%)			
Minutes	Cell wall	Cell interior	External EPS
30	56 ± 3	28 ± 5	16 ± 2
180	49 ± 1	49 ± 2	2 ± 1

The analysis of the parameters describing the persister-derived biofilm of C1 strain following the interaction with gH625-M are reported in Table 4. They clearly indicate that the biofilm was significantly damaged by the presence of the peptide. The effect was time-dependent: after 180 min, the substrate coverage, the biovolume, the mean and maximum thickness were appreciably reduced, whereas the roughness coefficient increased, indicating a structural damage of the biofilm, following the treatment.

We also analyzed by CLSM the ability of gH625-M to penetrate across the EPS of the biofilm and, eventually *Candida* cells. In particular, Fig. 5 (panels a and g) shows that the cell wall was not damaged by the treatment with the peptide both at 30 and 180 min (blue). A marked difference between the samples treated with Concanavalin-A at 30 min and 180 min can be observed (Fig. 5, panels b and h) (green); the green fluorescence is diffused around the cell after 30 min from the treatment, but after 180 min fluorescence is mainly located inside the cell; this result may indicate that the peptide was able to permeabilize the matrix and allow Concanavalin-A to enter inside the cell, where it could stain mannosyl and glucosyl groups inside the cell. This seems to be in agreement with the fact that gH625 is able to cross membrane bilayers without damaging them and without producing leakage and is able to favour the penetration of the cargos. In the same figure (panels c and i) (red) the localization of the peptide is shown; it is mainly located into the cell wall at 30 min, but at 180 min it is essentially located inside the cell. The other panels report a three dimensional reconstruction of a half single cell. Indeed, in Fig. 5 (panels d and j) it is possible to observe that the peptide is located on the cell wall after 30 min, and then inside the cell. Figure 5 (panels e and k) shows the change in the green fluorescence following the treatment, as compared to the control (panels f and l).

These images corroborate the data reported in Table 5 on the distribution of gH625-M in the biofilm (cell wall, cell interior and external matrix) after 30 and 180 min of exposure. After 180 min, the percentage of peptide in the cell interior is significantly enhanced, while that in the external EPS/matrix is reduced.

## Discussion

Persister cells are rare phenotypic variants found in susceptible microbial populations able to tolerate lethal concentrations of antimicrobials and to grow again after their removal, with an antimicrobial sensitivity equal to that of the original population. The ability of persisters to evade the antimicrobial treatment is one of the possible causes of recalcitrant infections; thus, it is important to understand the mechanisms giving rise to persister cells in order to find a more efficacious treatment for chronic infections. This study was aimed to ascertain the ability of persisters found in *C. albicans* biofilms of reviving in an *in vitro* model, and the possibility to eradicate the persister-derived biofilm with alternative strategies.

Therefore, in this work we initially demonstrated the capability of persisters from biofilms of two different *Candida* strains to re-colonize the surface of a multiwell plate, originating a new biofilm similar to the primary one, as far as it concerns the amount of live biomass. Moreover, the new biofilm resulted less compact, showing a diversification between the inner and the outer part likely due to the presence of dispersed aerial hyphae^49^; a characteristics that could favour the dispersion of new cells in the surroundings, consequently contributing to infection dissemination. Persisters are not mutants but phenotype variants of the wild type, and mechanisms of cell transition remain unclear. It has been previously proposed that a different regulation of genes correlated to ergosterol (*ERG1* and *ERG25*) and β-1,6 glucan (*SKN1* and *KRE1*) pathways is involved^52^. Furthermore, comparisons between the membranes of planktonic cells and membranes of cells in the biofilm, showed that the latter contained a lower concentration of ergosterol, mainly in the deepest layers of the biofilm; it is likely that less ergosterol is necessary to cells from mature biofilms for maintaining membrane fluidity and this is further supported by the limited efficacy of drugs targeting ergosterol. *C. albicans* biofilm matrix is responsible of sequestering drugs, acting as a sponge and conferring resistance^53,54^.

The antifungal polyene, AmphB, is fungicidal, intercalating into membranes containing ergosterol, producing pores that cause the release of cell contents^55^. Besides, fluconazole, possesses a fungistatic effect, blocking ergosterol synthesis, targeting the enzyme lanosterol 14α-demethylase (related to the *ERG11* gene) and leading to an accumulation of toxic sterol pathway intermediates^56^. 5-fluorocytosine is fungistatic towards *C. albicans*; in particular, it is converted into 5-fluorouracil inside cells and subsequently into the metabolite 5-fluorodeoxyuridine, which inhibits RNA and DNA synthesis. Thus, it is the only antifungal drug in use having a mechanism of action correlated to an interaction with nucleic acids^57^; unfortunately, the risk of adverse effects of development of secondary resistance is high; its clinical use as a single antifungal agent is problematic due to its relatively weak antifungal efficacy and fast development of resistance; thus, it is preferably used only in targeted therapy^58–60^.

The emergence of resistance and the low activity of some drugs correlated to their delivery especially in biofilms, makes it necessary to explore new strategies for the development of more effective therapies. In this context, great interest is devoted to the use of AMPs. The innovative approach of this study is the use of cell penetrating peptides (CPPs), bearing also some antimicrobial activity, and combined to conventional antifungal drugs. In fact, the use of CPPs could facilitate the crossing of the biofilm matrix and/or the cell envelopes by conventional drugs. For this reason, we selected a cell penetrating peptide, gH625, with the known ability of crossing membrane bilayers, which presented a relatively low activity against planktonic cells, but has already revealed a significant activity of biofilm eradication^33^.

In this study to test their eradication capability versus *Candida* biofilms, we used two peptides, namely gH625-M and gH625-D, which were previously designed in another study to dissect the role of multivalency in the ability of gH625 to cross membrane bilayers^34^. Comparisons between monomers (gH625-M) and dimers (gH625-D) confirmed that the mechanism of action involves a local perturbation of the membrane bilayer which enables the peptide to cross the membrane by a physical mediated mechanism not involving pore formation and enhancing the intracellular uptake^34^.

CPPs such as gH625 exhibit cross-functionality with some AMPs able to cross mammalian cell membranes by non-damaging processes, and several CPPs display significant antimicrobial activity^21^. Further, oligomerization improves the insertion of gH625 into a lipid bilayer and enhances its ability to cross the membrane bilayer, though we could not evidence a significant enhancement of the antibacterial activity correlated to oligomerization. This was also confirmed in this study by the high MIC values on planktonic *C. albicans* cells, thus demonstrating that gH625 whether monomeric or dimeric has a scarce antimicrobial activity.

Interestingly, the two analogues and especially the monomeric form were very efficient in eradicating mature *C. albicans* biofilms as well as persister-derived biofilms.

The elucidation of the higher activity in the eradication process can be found in the chemical nature of the peptide gH625, which is a viral derived sequence rich in hydrophobic residues. The previous characterization of gH625 has shown that it is able to interact with model membranes, penetrate in the lipid bilayer and induce fusion between membranes; moreover, the presence of the positive charges at the C-terminus may enhance the interaction with the negatively charged *Candida* membranes and/or the matrix of the *Candida* biofilm. Here, the CLSM analysis of the biofilm after different exposure times to gH625-M confirmed that the peptide was able initially to interact with the EPS, so to disrupt the biofilm organization and likely provoking its eradication, and subsequently also entered into the cells. gH625 does not have activity against planktonic cells because it does not form pores in the membrane and has not an intracellular target. On the contrary, it presents activity against the biofilm because it acts on the structure of the biofilm or quorum sensing and may enhance other drugs’ activities favouring their internalization or membrane interaction.

Further, we wanted to explore the ability of gH625-M alone or in combination with conventional drugs to combat persister-derived biofilms. The analysis of the FICI values clearly shows that there is a synergistic effect with all the drugs used in this study, both for those targeting membrane ergosterol (AmphB) and those with intracellular targets (fluconazole and 5-FC). 5-FC represents certainly the most interesting result because it exerts an antifungal activity only after its uptake by fungal cells. Our results clearly show that it has basically no activity when used alone, whereas the combined use of 5-FC and gH625-M produces a significant increase in its antifungal activity. As already mentioned, the mode of action of the drugs used and of gH625-M are extremely different. The major mechanism of action of gH625-M is initially directed towards the EPS and then towards cell membranes, producing a local and temporary destabilization, which probably facilitates the entrance of conventional drugs, promoting a synergistic activity that may affect also targets inside the cells. Likely, the 5-FC low fungicide activity results from difficulties of this molecule to enter the cells, which leads to a decrease of activity. On the other hand, gH625-M targets the membrane and favours the crossing of the membrane of any cargo, which is covalently or non-covalently bound.

## Conclusions

Our results are opening up a wide range of possibilities correlated to the use of gH625 and its analogues as antibiofilm molecules in combination with conventional drugs. In particular, in the scenario of possible exploitations of the translational potential of gH625, we could consider to use this peptide both to destroy a pre-existing biofilm and as carrier of other anti-infective agents for a synergistic effect. Future studies are needed to better understand the mechanism of action on the biofilm and to extrapolate our results to other microrganisms and are going on in our laboratory.
